# Unraveling angioedema: diagnostic challenges and emerging therapies

**DOI:** 10.3389/fimmu.2025.1681763

**Published:** 2025-10-01

**Authors:** Felix Johnson, Benedikt Hofauer

**Affiliations:** University Hospital for Otorhinolaryngology, Head and Neck Surgery, Medical University of Innsbruck, Innsbruck, Austria

**Keywords:** bradykinin, angioedema, hereditary angioedema, urticaria, ACE inhibitor angioedema

## Abstract

Bradykinin-mediated angioedema comprises rare but potentially life-threatening disorders, most notably hereditary angioedema (HAE) due to C1 inhibitor (C1-INH) deficiency or dysfunction. Diagnosis is often difficult, as these conditions can resemble urticaria variants, leading to misdiagnosis and delays in care. Distinguishing features are critical, since bradykinin-mediated forms do not respond to antihistamines or corticosteroids. This review summarizes the differential diagnoses of angioedema, including urticaria variants, cheilitis granulomatosa, and hypocomplementemic urticarial vasculitis, highlighting clinical and diagnostic clues. Particular focus is given to HAE—its subtypes (Type I, Type II, and normal C1-INH), pathophysiology, presentation, and genetic basis. Acquired angioedema and drug-induced forms, such as ACE inhibitor–associated angioedema, are also discussed. The therapeutic landscape is rapidly evolving, spanning acute and prophylactic approaches. Options include C1-INH concentrate, kallikrein inhibitors, bradykinin receptor antagonists, and factor XII inhibitors. While these advances expand treatment opportunities, they also complicate decision-making for patients and physicians. Furthermore, emerging CRISPR-based gene editing therapies represent innovative approaches that pose complex ethical dilemmas, and their long-term safety and efficacy have yet to be established. Although novel therapies reduce attack frequency, their true impact on quality of life is not fully established. Comparative effectiveness data are limited, long-term safety—particularly of gene-based therapies—is unknown, and the real-world utility of new oral on-demand agents for acute therapy is uncertain, especially in severe pharyngeal or laryngeal attacks that may hinder swallowing. Current guidelines remain unclear on the need for short-term prophylaxis in patients already receiving effective long-term prophylactic therapy. In conclusion, despite major therapeutic advances, persistent challenges and unanswered questions underscore the need for pragmatic, patient-centered, long-term studies to optimize care.

## Introduction

Angioedema is characterized by localized swelling of the skin or mucous membranes resulting from fluid extravasation. It can arise through distinct mechanisms, most commonly histamine-mediated or bradykinin-mediated pathways, both of which involve vasodilation and increased vascular permeability. Histamine-mediated angioedema is typically accompanied by pruritus and erythema and usually responds well to antihistamines and glucocorticoids. In contrast, bradykinin-mediated angioedema lacks pruritus and erythema, does not respond to standard allergy-directed therapies, and instead results from excessive bradykinin activity leading to increased vascular permeability.

Bradykinin-mediated angioedema encompasses several clinically relevant subtypes, including hereditary angioedema (HAE), acquired angioedema, and drug-induced forms. Among these, HAE is particularly significant because of its genetic basis, potential for life-threatening attacks, and the availability of disease-specific targeted therapies. Importantly, the prognosis for patients with HAE is substantially improved with highly effective on-demand and prophylactic treatments, which dramatically reduce morbidity and mortality and significantly improve quality of life.

Accurate distinction between histamine- and bradykinin-mediated angioedema is therefore essential to guide appropriate management and avoid delays in effective treatment. This review provides an overview of the differential diagnosis, classification, and evolving therapeutic landscape of bradykinin-mediated angioedema, with a particular focus on HAE and related conditions.

## Discussion

### Differential diagnoses of bradykinin-mediated angioedema urticaria (hives)

The lifetime prevalence of urticaria is approximately 20%. It is based on a mast cell–mediated reaction and may present with wheals and/or angioedema. Wheals are pruritic, erythematous, elevated swellings in the superficial layers of the skin, whereas angioedema affects deeper layers and typically occurs without pruritus or erythema. Triggers include stress, infections, hormonal fluctuations, alcohol consumption, and nonsteroidal anti-inflammatory drugs (NSAIDs).

Urticaria is classified based on the duration of symptoms into acute (< 6 weeks) and chronic (> 6 weeks) forms. Chronic spontaneous urticaria (CsU) occurs either without identifiable triggers or in response to specific stimuli (inducible urticaria), which can be further subdivided into distinct subtypes (see **Table 1**). Pressure Urticaria causes edema in the deeper layers of the skin (1), typically occurring hours after the application of static pressure, such as prolonged sitting. Pruritus or erythema is often absent, making it an important differential diagnosis of hereditary angioedema.

**Table 1:** Classification of chronic urticaria.

Chronic spontaneous urticaria	Inducible urticaria
Unknown Etiology	Symptomatic Dermographism (formerly: Urticaria Factitia)
Known Etiology	Pressure Urticaria
	Vibratory Urticaria
	Cold Urticaria
	Heat Urticaria
	Light Urticaria
	Solar Urticaria
	Contact Urticaria

Chronic urticaria is divided into chronic spontaneous urticaria, where symptoms occur without an identifiable trigger, and chronic inducible urticaria, where specific stimuli provoke symptoms (e.g., pressure, temperature, light, or contact). This classification is clinically relevant as it guides diagnostic evaluation, patient counseling, and therapeutic strategies.

A thorough medical history—ideally supported by photographic documentation—is usually sufficient to establish the diagnosis of urticaria, and this can usually be confirmed by therapeutic response.

### Vibratory angioedema

This rare form of physical urticaria characterized by development of hives and localized swelling in response to vibratory stimuli. Vibratory angioedema may occur sporadically, but familial cases follow an autosomal dominant pattern and often present in childhood. Recent insights have linked hereditary vibratory angioedema to mutations in the ADGRE2 gene, which encodes an adhesion G protein–coupled receptor on mast cells (also known as EMR2) (2, 3). ADGRE2 is synthesized as a single polypeptide that is autoproteolytically cleaved into an extracellular α subunit and a transmembrane β subunit, which remain noncovalently attached under resting conditions. The pathogenic ADGRE2 variant (p.C492Y) weakens this α–β interaction, so that even mild mechanical vibration readily dissociates the α subunit, triggering the receptor’s signaling function. This aberrant mechanotransduction activates a cascade in mast cells involving G-protein–mediated phospholipase C signaling, calcium influx, and downstream kinases (PI3K, ERK1/2), leading to exaggerated degranulation and the release of histamine and prostaglandin D_2_. From a clinical standpoint, identifying an ADGRE2 mutation confirms the diagnosis of familial vibratory angioedema, but treatment remains focused on counteracting mast-cell mediators (3).

### Treatment of urticaria-mediated angioedema

First-line therapy for vibratory angioedema consists of high-dose second-generation H1-antihistamines (often in combination with H2 blockers and leukotriene antagonists), and refractory cases may benefit from an off-label therapy with omalizumab. These mechanistic insights underscore that vibratory angioedema is fundamentally a mast cell–activation disorder, highlighting its clinical overlap with other mast cell–mediated angioedema forms and distinguishing it from bradykinin-driven forms.

The primary treatment of urticaria consists of avoiding known triggers. Second-generation antihistamines are the mainstay of therapy and can be taken as needed in cases of infrequent attacks.

Early administration at the onset of prodromal symptoms such as tingling may prevent a full-blown episode. In cases of frequent attacks, the dosage may be increased off-label to up to four times daily. If the response to antihistamines is inadequate, treatment with omalizumab (an anti-IgE monoclonal antibody) is a therapeutic option (1).

Dupilumab has been evaluated in phase III trials for chronic spontaneous urticaria, where treatment was compared only to placebo. The studies included two patient populations: individuals naive to omalizumab, who demonstrated a marked clinical and quality-of-life benefit, and those with prior omalizumab intolerance or incomplete response, in whom effects were modest. Dupilumab demonstrated a −8.5-point reduction in Urticaria Activity Score over 7 days (UAS7) and a −4.2-point reduction in Itch Severity Score (ISS7) versus placebo in omalizumab-naïve patients; in omalizumab-intolerant or incomplete responders, only modest benefits were observed (UAS7 −5.8 points, borderline significance; ISS7 not significant) (4). Safety data indicate conjunctivitis and injection-site reactions as the most frequent adverse events. Importantly, long-term safety is supported by extensive experience from other indications. Since its initial EMA approval in 2017 for atopic dermatitis, followed by asthma and chronic rhinosinusitis with nasal polyps, dupilumab has established a favorable safety profile, which is relevant when considering its role in CSU. Additional real-world and comparative studies are needed to better define its place in treatment algorithms.

Phase IIb studies on oral prophylaxis of chronic spontaneous urticaria with remibrutinib, a Bruton’s tyrosine kinase (BTK) inhibitor, have demonstrated convincing efficacy with minimal adverse effects (absolute risk reduction [ARR] at 10 mg once daily: 19.1-point reduction in Urticaria Activity Score) (5, 6). In Phase III REMIX-1 and REMIX-2 trials, remibrutinib induced rapid and sustained reduction in urticaria activity (UAS7), with significant improvements also seen in itch severity (ISS7), hives severity (HSS7), and overall disease control as quantified by the Urticaria Control Test (UCT7) (7).

In terms of comparative efficacy, no direct trials against antihistamines, omalizumab or dupilumab are available, leaving its relative positioning uncertain. Cost-effectiveness analyses are still lacking, and long-term durability data remain limited. Patient-reported outcomes were a strong component of the pivotal studies, but real-world data are not yet available. The strength of evidence is therefore high for short-term efficacy but weaker for long-term outcomes. The risk of adverse effects were almost identical in the placebo and remibrutinib groups (64.7% and 64.9% respectively) with headache and mild gastrointestinal events most frequently reported (7).

Barzolvolimab inhibits mast cell activation in the treatment of cold urticaria and symptomatic dermographism. Phase II results of barzolvolimab have been reported as top-line findings, demonstrating significant reductions in urticaria activity scores and sustained efficacy, but full peer-reviewed data with detailed subgroup and safety analyses remain pending. This randomized, placebo-controlled, double-blind study included patients who were refractory to antihistamines and was conducted over a twelve-week period. In cold urticaria, the ARR in the 150 mg dose group was 34.4%, corresponding to a number needed to treat (NNT) of approximately 3. In the 300 mg group, the ARR was 40.6% (NNT ~2.5).

In symptomatic dermographism, where mechanical irritation of the skin via pressure or shear forces provokes symptoms, even greater efficacy was observed: the ARR was 54.4% in the 150 mg group (NNT ~2) and 39.2% in the 300 mg group (NNT ~2.5). The most common adverse events reported were hair discoloration, neutropenia, and dysgeusia (8). Hair color changes were reported in approximately 26% of patients at 52 weeks; these effects were reported as mild and reversible after treatment cessation (9). Cost considerations and long-term safety remain undefined.

### Cheilitis granulomatosa (orofacial granulomatosis)

Clinically, the disease presents with swelling of the upper lip, lower lip, or cheeks. Initially, symptoms occur in episodes, but over time the disease tends to become chronic (10). Flares can last for weeks or even months and are typically much more prolonged than angioedema attacks.

### Hypocomplementemic urticarial vasculitis syndrome

Urticarial vasculitis is characterized by complement deficiency, commonly accompanied by anti C1q antibodies and urticarial skin lesions. The cutaneous manifestations often persist significantly longer than those of ordinary urticaria (i.e., more than 24 hours), appear erythematous, and are pruritic. When deeper vessels are involved, angioedema may also occur (11).

### Urticarial vasculitis

Urticarial vasculitis presents with erythematous, raised skin lesions that resemble urticaria but are often painful or burning rather than pruritic, and persist for more than 24 hours. These lesions typically resolve with residual hyperpigmentation or purpura, distinguishing them from transient, histamine-mediated urticaria. Angioedema may accompany the rash, sometimes mimicking allergic or bradykinin-mediated forms. Systemic features such as arthralgia, low-grade fever, and abdominal pain may occur, particularly in hypocomplementemic subtypes, which are often associated with autoimmune disease or more extensive organ involvement (12).

### Cryoglobulinemia

Cryoglobulinemia-associated vasculitis may manifest with urticarial or purpuric skin lesions, often located in dependent or cold-exposed areas. These are typically painful, may persist for several days, and can resemble urticarial vasculitis or angioedema in their initial presentation. Systemic symptoms such as arthralgia, peripheral neuropathy, and renal impairment are common, and complement levels are frequently reduced. The presence of circulating cryoglobulins, often in the setting of hepatitis C infection or lymphoproliferative disease, supports the diagnosis (13).

### Schnitzler syndrome

Schnitzler syndrome is an autoinflammatory condition characterized by a chronic, nonpruritic urticarial rash, which may be mistaken for chronic spontaneous urticaria due to its appearance. However, the lesions are typically persistent, lack the migratory nature of histamine-induced wheals, and are accompanied by systemic features including recurrent fever, bone pain, and arthralgia. Some patients also report swelling resembling angioedema, although it is not a defining feature. The syndrome is associated with a monoclonal IgM gammopathy and shows a characteristic response to interleukin-1 inhibition (14).

### Bradykinin-mediated angioedema

Subtypes of bradykinin-mediated angioedema include hereditary angioedema (HAE), acquired angioedema, idiopathic angioedema, angiotensin-converting enzyme (ACE) inhibitor– or angiotensin receptor blocker (ARB)–induced angioedema, and thrombolysis-associated angioedema. Although these conditions may differ in their underlying pathomechanisms, they share a common final pathway: elevated bradykinin levels. Angioedema attacks do not respond to antihistamines or glucocorticosteroids.

### Hereditary angioedema: classification and diagnosis

**Figure 1:**
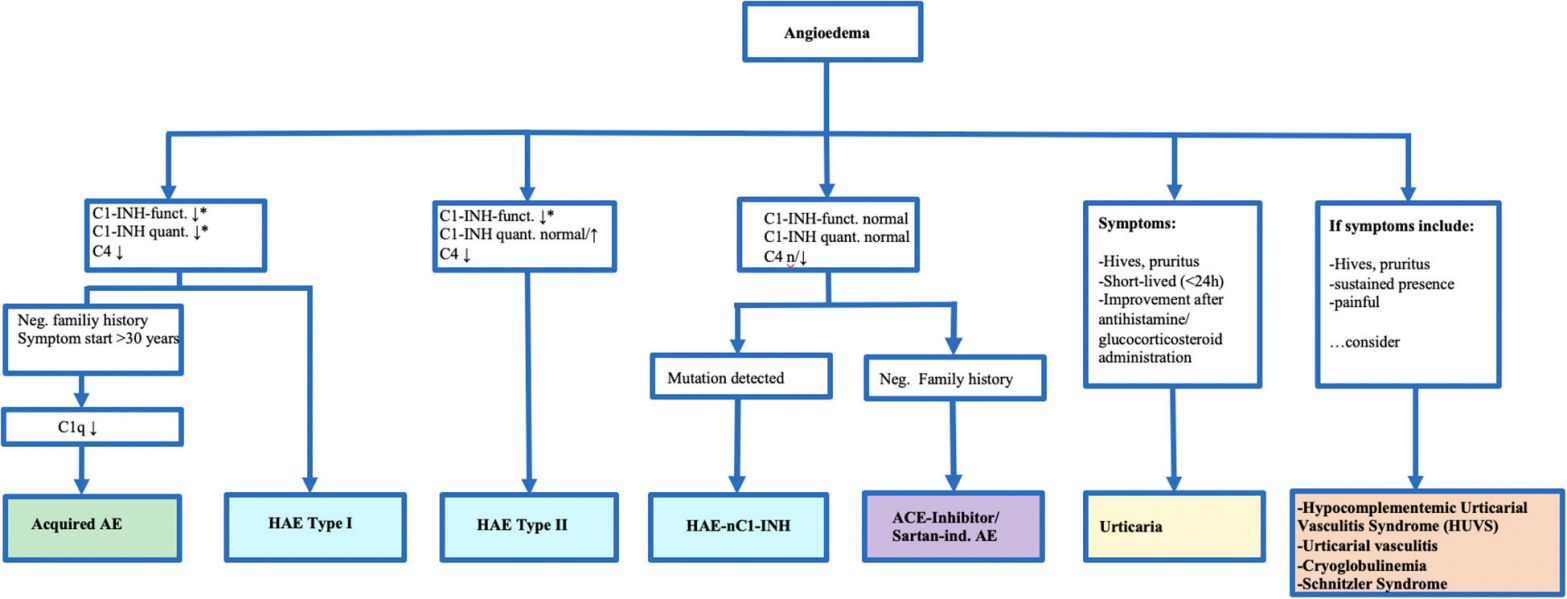
Diagnostic and Classification of Bradkinin-Induced Angioedemas and Relevant Differential Diagnoses. *Indicates that values are typically reduced to about 50% of normal levels in affected patients. This algorithm highlights the diagnostic pathway for distinguishing hereditary and acquired angioedema subtypes based on Cl-inhibitor (C1-INH) function and quantity, clinical features, and family history. The figure is clinically relevant as it guides physicians through the main steps of differentiating hereditary angioedema (HAE) type I, type II, and HAE with normal C1-INH from other forms of angioedema, as well as distinguishing bradykinin-mediated angioedema from histamine-mediated urticaria and related differential diagnoses.

Hereditary angioedema (HAE) refers to a group of rare orphan diseases (incidence: 1 in 50,000) with overlapping pathophysiology, treatment strategies, and clinical presentation. HAE is classified into Type I, Type II, and HAE with normal C1-esterase inhibitor levels (HAE-nC1-INH) (see **Figure 1**). HAE is inherited in an autosomal dominant manner, meaning that affected parents have a 50% chance of passing the condition on to their offspring. In HAE due to C1-INH deficiency (Types I and II), up to 25% of cases arise from *de novo* mutations. Thus, while a positive family history can aid in diagnosis, its absence does not rule out the disease. By contrast, HAE with normal C1-INH is most often familial, and *de novo* cases are not well documented (15).

**Figure 2:**
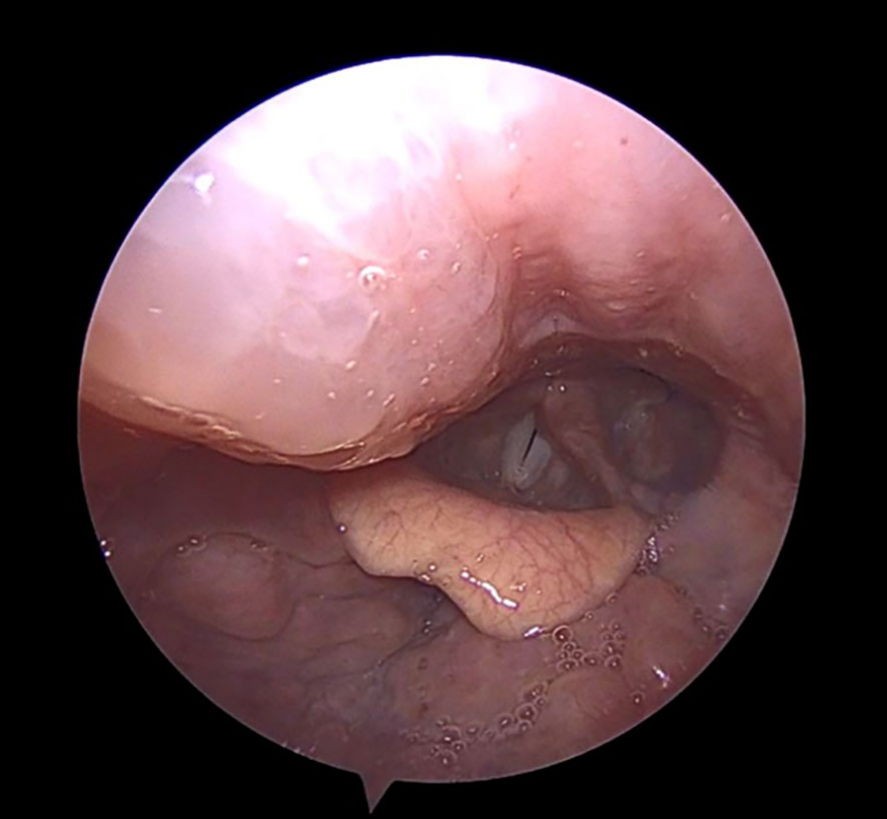
Hereditary angioedema (HAE) attack with involvement of the right posterior pharyngeal wall descending towards the glottis (appearing on the left side of the image). HAE attacks involving the upper airway may be life-threatening. Patients presenting with pharyngeal or laryngeal swelling should ideally be managed in an otorhinolaryngology center with the capacity for close airway monitoring and, if required, prompt intubation or surgical airway protection.

Clinically, HAE presents with recurrent, non-pruritic, non-erythematous swellings of the skin and mucous membranes that can affect any part of the body, including the gastrointestinal tract, which may be extremely painful and lead surgeons to suspect an acute infection of gastrointestinal tract. Airway swelling is potentially life-threatening as seen in **Figure 2**, which illustrates a pharyngeal swelling which may lead to airway obstruction. Mortality rates due to laryngeal angioedema alone average 5% in the HAE population (16), therefore, any suspected pharyngeal or laryngeal attack should be treated without delay and monitored in a clinical setting—ideally in an otorhinolaryngology clinic equipped for airway management. In some cases, emergency intubation or even surgical airway intervention may be required.

Angioedema symptoms typically begin around puberty, with about half of patients experiencing their first symptoms before the age of 10 (17). HAE attacks can be triggered by hormonal changes, stress, infections, trauma, surgical procedures, or medications such as ACE inhibitors (18).

### HAE type I and II

Researchers estimate that about 85–90% of patients have HAE type I, while 10–15% have HAE type II. In HAE Type I, there is reduced production of C1-esterase inhibitor (C1-INH), whereas in Type II, the enzyme is produced but functionally defective. This enzyme is the sole inhibitor of bradykinin production; its deficiency or dysfunction leads to excessive bradykinin generation and the development of edema. Diagnosis of HAE is based on typical clinical features in combination with a C1-esterase inhibitor concentration or functional activity below 50% of normal. In cases with inconclusive findings or rare variants, genetic testing can help identify pathogenic mutations (19).

### Normal C1-INH (HAE-nC1-INH)

Though it is estimated that HAE-nC1-INH comprises less than 5% of all HAE forms, this number be higher. In recent years, multiple pathogenic mutations have been identified in HAE-nC1-INH, defining distinct molecular subtypes. Currently six different variants have been described. The most common involve the F12 gene (coagulation factor XII), where gain-of-function variants render FXII more susceptible to activation, precipitating kallikrein–bradykinin excess under hormonal triggers. These patients are predominantly female and disease activity is associated with increased estrogen levels (20, 21). Mutations in PLG create plasmin with altered catalytic activity that acquire the ability to cleave high-molecular-weight kininogen, resulting in bradykinin release independent of the kallikrein pathway; KNG1 variants alter high–molecular-weight kininogen; ANGPT1 mutations impair the endothelium vascular stabilizer angiopoietin 1; and MYOF mutations likely affect endothelial integrity; and HS3ST6 leads to biosynthesis of heparan sulfate, a crucial molecule for vascular integrity and endothelial barrier function (19, 22). Researchers suspect that many cases remain genetically unexplained. Diagnostic workup now often includes targeted gene sequencing panels for these variants, since normal C1INH in the presence of recurrent angioedema suggests this diagnosis.

### Acquired angioedema (AAE-C1-INH)

Acquired angioedema with C1-esterase inhibitor deficiency (AAE-C1-INH) has an incidence of approximately 0.15 cases per 100,000 individuals. AAE-C1-INH arises from accelerated degradation of C1-esterase inhibitor and is often linked to lymphoproliferative disorders or autoantibody-mediated complement dysregulation. Because these underlying conditions may be malignant, AAE-C1-INH presents distinct clinical and therapeutic challenges that necessitate careful management of both the angioedema and the primary disease. The angioedema attacks are a clinical manifestation of the underlying condition and are indistinguishable from those observed in HAE. AAE-C1-INH should be suspected in patients with a first episode of angioedema after age 30, particularly in the absence of a family history. The median age at diagnosis is 65 years, distinguishing it from hereditary forms, which typically present earlier in life. Laboratory findings show reduced C1-INH function. A key diagnostic marker is decreased C1q, observed in approximately 70–80% of AAE-C1-INH patients, whereas C1q levels are typically normal in HAE. However, up to 20-30% of patients lack detectable anti-C1-INH antibodies despite clinical and laboratory features consistent with AAE, suggesting that other mechanisms may contribute to C1-INH deficiency in a subset of patients (23). Autoantibodies, typically of the IgG1 or IgG4 subclass, interfere with both antigenic and functional C1-INH assays. Commercial testing is limited, and antibody titers do not consistently correlate with disease activity, complicating diagnosis and therapeutic monitoring (24).

### Idiopathic bradykinin-mediated angioedema

Idiopathic bradykinin-mediated angioedema is a rare diagnosis of exclusion, considered only after histamine-mediated conditions, hereditary angioedema, and acquired angioedema have been ruled out. Diagnostic confirmation can be supported by therapeutic trials using agents such as icatibant or C1-esterase inhibitor. Idiopathic bradykinin-mediated angioedema is considered much rarer than drug-mediated forms (e.g., ACE-inhibitor–associated angioedema), but robust epidemiological data on its incidence or prevalence are lacking.

### Thrombolysis-mediated angioedema

In acute cerebral ischemia, thrombolysis with recombinant tissue plasminogen activator (rt-PA) may be performed. A known adverse effect of this treatment is increased bradykinin production. The prevalence of post-thrombolysis angioedema is estimated to be between 1% and 2%, and often causes swellings of the tongue (25, 26).

### Angiotensin-converting enzyme-inhibitor- and sartan-mediated angioedema

ACE inhibitors and sartans are widely used, and angioedema is a well-recognized complication often seen in clinical practice. They can cause elevated bradykinin levels, resulting in angioedema that typically develops approximately three years after the initial dose. The incidence of bradykinin-mediated angioedema ranges for ACE-inhibitors between 0.2% to 0.7%, with a roughly fivefold increased risk observed in patients with darker skin pigmentation (27). Sartan-mediated angioedema is significantly less common (0.11%) (28).

### Treatment of bradykinin-mediated angioedema

Management of angioedema attacks in patients with hereditary angioedema (HAE) (see **Table 2**) involves three primary strategies: on-demand therapy, short-term prophylaxis, and long-term prophylaxis. The choice of treatment is guided by the severity and frequency of attacks, as well as individual patient circumstances.

**Table 2:** Medications approved for HAE.

Medication	Indication	Delivery	Dosage in mg (adults)	Age (availability, EU)	Price in Germany (€)
On-Demand			Body weight (75kg)		1x Administration
Icatibant	Acute HAE Attacks	s.c.	30 mg	From 2 years (EU)	2,610.97 (Generic: 472.49)
C1-INH (human) (Berinert)	Acute HAE Attacks, Short-Term Prophylaxis*	i.v.	20 IE/kg body weight *1000IE	From 0 years (EU)	2,638.56 (*1,759.04)
C1-INH (human) (Cinryze)	Acute HAE Attacks, Short-Term Prophylaxis	i.v.	1000 IE	From 6 years (EU)	2,240.00
C1-INH (recombinant)	Acute HAE Attacks	i.v.	50 IE/kg body weight	From 2 years (EU)	2,592.20
Prophylaxis			Body weight (75kg)		Administration over 1 year
C1-INH (human) (Berinert)	Long-Term Prophylaxis	i.v.	20 IE/kg body weight	From 12 years (EU)	253,301.76
C1-INH (human) (Berinert)	Long-Term Prophylaxis	s.c.	60 IE/kg body weight (2x per week)	From 12 years (EU)	646,970.88
Lanadelumab	Long-Term Prophylaxis	s.c.	300 mg (every 2–4 weeks)	From 2 years (EU)	258,087.24 (2 weeks)
Berotralstat	Long-Term Prophylaxis	p.o.	150 mg (daily)	From 12 years (EU)	206,530.16
C1-INH (human) (Cinryze)	Long-Term Prophylaxis	i.v.	1000 IE (2x per week)	From 6 years (EU)	207,675.84
Garadacimab	Long-Term Prophylaxis	s.c.	(every 4 weeks)	From 12 years (EU)	374,780

Prices shown reflect the German pharmacy retail price (Apothekenverkaufspreis, AVP, including Value Added Tax) at the time of writing. As medication costs vary considerably between countries and healthcare systems, the listed values are intended for illustrative purposes only and to provide context on the relative cost burden of different therapies, rather than for direct international comparison.

### On-demand therapy

This approach is used to promptly treat acute attacks and alleviate symptoms. Therapeutic options include C1-esterase inhibitor concentrate, bradykinin B2 receptor antagonists (e.g., icatibant), and kallikrein inhibitors. In emergency cases where other treatments are not available fresh frozen plasma may be administered.

Icatibant is effective as on-demand treatment and can quickly be administered subcutaneously, with registry data supporting rapid symptom control when administered early. Comparative analyses suggest similar efficacy to C1-inhibitor concentrate, although retreatment rates may be higher in some patients due to rebound attacks. Cost-effectiveness depends largely on timing of administration and the need for repeat doses due to rebound attacks. Injection-site reactions are the most common adverse effect. The overall evidence is robust, supported by large real-world registries (29).

### Short-term prophylaxis

Short-term prophylaxis aims to prevent attacks in high-risk situations, such as surgical procedures, dental work, or other traumatic events. It typically involves administering C1-esterase inhibitor concentrate approximately one hour prior to the intervention. Current guidelines do not clearly define whether short-term prophylaxis is necessary in patients who are already receiving highly effective long-term prophylactic therapy.

### Long-term prophylaxis

According to the German guideline for hereditary angioedema, long-term prophylaxis should be considered in patients with frequent or severe attacks, insufficient response to acute therapy, or a high risk of medical emergencies. Improving quality of life is also a key consideration in initiating long-term therapy as recommended in the international WAO/EAACI guideline for the management of hereditary angioedema (19).

Long-term treatment options include C1-esterase inhibitor concentrates, kallikrein inhibitors, or factor-XII inhibitors. Androgens, once widely used for long-term prophylaxis in hereditary angioedema (HAE), are no longer recommended as first-line therapy. This shift reflects the availability of safer, better-tolerated alternatives and the considerable risk of adverse effects associated with androgen use, including virilization, hepatotoxicity, and hepatocellular carcinoma (30).

Intravenous administration of plasma-derived C1-esterase inhibitor (C1-INH) concentrate was approved in 1979 and is considered a safe and reliable treatment for acute HAE attacks, including during pregnancy (31). There is no defined age restriction, and pediatric studies have also confirmed its safety and efficacy (32). In a six-month study, intravenous plasma-derived (nanofiltered) C1-INH concentrate significantly reduced the mean number of HAE attacks (6.1 ± 5.4 attacks) compared to placebo (12.7 ± 4.8 attacks) (33).

Subcutaneous (s.c.) plasma-derived C1-INH concentrate, available in 2000 IU and 3000 IU doses, has been approved for prophylactic use since 2018. In a 16-week randomized, placebo-controlled trial, a median reduction in attack frequency of 95% was observed compared to placebo, with an absolute risk reduction (ARR) of 2.84 attacks per week (34). Despite its robust efficacy, it is considerably more expensive than other injectable prophylactic options (see **Table 2**).

Lanadelumab is a monoclonal antibody targeting plasma kallikrein, approved since 2019 for s.c. prophylaxis of hereditary angioedema. The drug has a well-established efficacy and safety profile. After six months, the monthly attack rate was reduced by 96.6% in the group receiving 300 mg every four weeks, compared to a 20.8% reduction in the placebo group. The absolute risk reduction (ARR) for the 300 mg every two weeks regimen was 1.71 attacks per month (35, 36).

In the an open-label extension (up to 30 months), attack rates were reduced by 87%, with patients remaining attack-free for 98% of days, and AE-QoL scores showed improvement within the first months of treatment and these improvements were maintained through 30 months of follow-up (37).

In clinical practice, many patients begin on the every-2-week regimen, and if they remain well controlled, clinicians extend the interval to every 4 weeks. In a real-world study, attack frequency reduction remained favorable even when patients were switched to longer dosing intervals, indicating that 4-week administration was successful for a subset of patients without loss of efficacy (38).

Garadacimab is a first-in-class monoclonal antibody that inhibits activated factor XII and is intended for monthly s.c. prophylaxis. In Phase III studies, garadacimab demonstrated excellent efficacy with an ARR of 1.74. A randomized, double-blind, placebo-controlled trial reported an average reduction of attacks by 87% after six months of treatment. Patients achieved meaningful improvements in AE-QoL scores, with up to 88–92% exceeding the minimal important difference threshold. The most frequently reported adverse events were nasopharyngitis and injection-site reactions, which were generally mild (39, 40). The available evidence base is strong for short-term efficacy, but long-term safety remains to be established. There is almost no published real-world data, as garadacimab was only approved in spring 2025 for patients aged ≥12 years. Its once-monthly administration may offer greater convenience than more frequent injectable regimens. Comparative effectiveness and cost-effectiveness analyses are lacking, and the absence of head-to-head data against established prophylactic agents such as lanadelumab.

Berotralstat, a kallikrein inhibitor, is the only approved oral long-term prophylaxis for hereditary angioedema in patients 12 or older. After six months of treatment with berotralstat, there was an average reduction in attack frequency of 66.7% compared to baseline (absolute risk reduction [ARR] at 150 mg: 1.04 attacks/month). After 24 months, the attack rate was reduced by an average of 90.1% relative to baseline. Patient-reported outcomes highlight improved convenience and satisfaction with an oral option, with improvements in angioedema quality-of-life (AE-QoL) scores in trials and real-world studies, though real-world data is limited (41–43). Comparative effectiveness data remain limited: no head-to-head trials against lanadelumab or s.c. C1 inhibitor have been performed, and indirect comparisons suggest somewhat slower onset of maximal efficacy compared to injectable prophylaxis. It is generally well tolerated, although gastrointestinal adverse events are frequent, particularly at treatment initiation, though can often be mitigated by dose escalation. Interim results evaluating safety and efficacy in pediatric cases have delivered similar results to the adult population (44). In the EU, marketing authorization was granted on April 30, 2021, providing several years of experience with a safety profile consistent with trials and long-term extensions.

Sebetralstat is an oral plasma kallikrein inhibitor approved by the European Medicines Agency (EMA) in July 2025, with broader market availability and additional regulatory decisions anticipated soon. It has been approved for the on-demand treatment of acute hereditary angioedema attacks in adults and adolescents aged 12 years and older. Administered as two 300 mg tablets (600 mg total) at the earliest sign of an attack, sebetralstat offers a non-injection-based option for acute attack management. In the Phase III trial, it demonstrated rapid symptom relief, with a median time to onset of effect of 7.75 hours for the 600 mg dose, compared to over 12 hours for placebo. The 600 mg dose also resulted in a 22.1% reduction in attack severity compared to placebo. Reported adverse events were generally mild, including headache and gastrointestinal discomfort. As a swallowed oral medication, gastrointestinal side effects may be more prominent compared to s.c. therapies. Patient-reported outcomes were not extensively reported, and direct comparative data versus existing on-demand therapies, such as icatibant or C1 inhibitors, are lacking. Real-world performance remains uncertain, particularly for severe pharyngeal or laryngeal attacks, where swallowing may be compromised; sebetralstat must be swallowed and is not sublingual, which may limit use in some emergency situations. Overall, evidence supports strong efficacy in controlled trials, but gaps remain regarding its performance in high-risk real-world scenarios, pediatric populations, and long-term safety (45).

### Treatment of HAE with normal C1-INH

Treatment of HAE with normal C1-INH (HAE-nC1-INH) is centered on inhibition of the bradykinin pathway, although therapeutic responses are more heterogeneous than in classical HAE. In HAE-FXII, on-demand treatment with icatibant or plasma-derived C1-INH is effective in many cases; however, limited benefit was reported in up to 20% of patients treated with pdC1-INH, and relapse after initial control occurred in over half of patients treated with icatibant (46). In contrast, HAE-PLG patients generally responded well to the same therapies, with most reports indicating good efficacy. Although formal trials are lacking in HAE-nC1-INH, the use of lanadelumab has shown promising results in reducing attack frequency among patients with FXII and PLG mutations (47). Avoidance of known triggering factors (estrogens, ACE-inhibitors) is advised. Some subtypes of HAE-nC1-INH—particularly those with ANGPT1 or MYOF mutations—may not benefit from bradykinin-targeted therapy such as C1-INH concentrate or icatibant, underscoring the need for genotype-guided treatment decisions and ongoing research into alternative pathogenic pathways.

### Treatment during pregnancy, delivery, and breast-feeding

The clinical symptoms of HAE may be aggravated during pregnancy. The greatest clinical experience in managing HAE during pregnancy or breastfeeding is with intravenously administered plasma-derived C1-esterase inhibitor (pdC1-INH), which can be used for both on-demand treatment and long-term prophylaxis. Evidence indicates that pdC1-INH is effective and safe in this setting. Real-world data indicate that icatibant is effective and generally well tolerated; however, HAE guidelines recommend its use only when pdC1-INH is unavailable (19, 48). With respect to delivery, short-term prophylaxis is not generally required for vaginal birth. In contrast, for cesarean section, prophylactic administration is recommended (49). Assessment of C1-inhibitor levels using umbilical cord blood is not recommended. Delivery should take place in a hospital equipped to provide immediate access to appropriate HAE therapies.

### Treatment of acquired angioedema (AAE-C1-INH)

In suspected cases of AAE-C1-INH, hematologic and oncologic evaluation is recommended. Often, angioedema attacks are symptoms of low-grade malignancies that may not require immediate treatment. In such cases, clinical observation may be sufficient, as systemic therapy can carry significant potential side effects. Treatment of the underlying disease may improve symptoms but does not necessarily result in reduced angioedema attacks. Management focuses on treating any underlying disorder and applying prophylactic strategies extrapolated from hereditary angioedema. However, B-cell–depleting therapy with rituximab (often combined with chemotherapy) has been shown to induce remission in many patients; in one multicenter study, approximately two-thirds of cases achieved attack-free status following rituximab-based treatment (50, 51).

While established treatment guidelines and numerous clinical trials exist for HAE, specific guidelines and large-scale studies for AAE-C1-INH are lacking, and symptomatic treatment of angioedema attacks is off-label.

Smaller cohort studies suggest that therapeutic approaches effective in HAE—such as lanadelumab, berotralstat, icatibant, or plasma-derived C1-INH concentrate—may also be effective off-label treatment options for AAE-C1-INH (43, 52, 53). Among newer targeted options, oral kallikrein inhibition with berotralstat (150 mg daily) nearly halved attack frequency in patients with AAE (median 2.3 to 1.0 attacks per month) and significantly improved quality of life in a recent retrospective cohort (43). Unlike plasma-derived C1-INH, this strategy is not vulnerable to inactivation by circulating antibodies. Similarly, a small cohort study supported the use of lanadelumab for long-term prophylaxis in AAE with consistently reduced attacks over 4 years of treatment (54).

### Treatment of angiotensin-converting enzyme-inhibitor- and sartan-mediated angioedema

**Figure 3:**
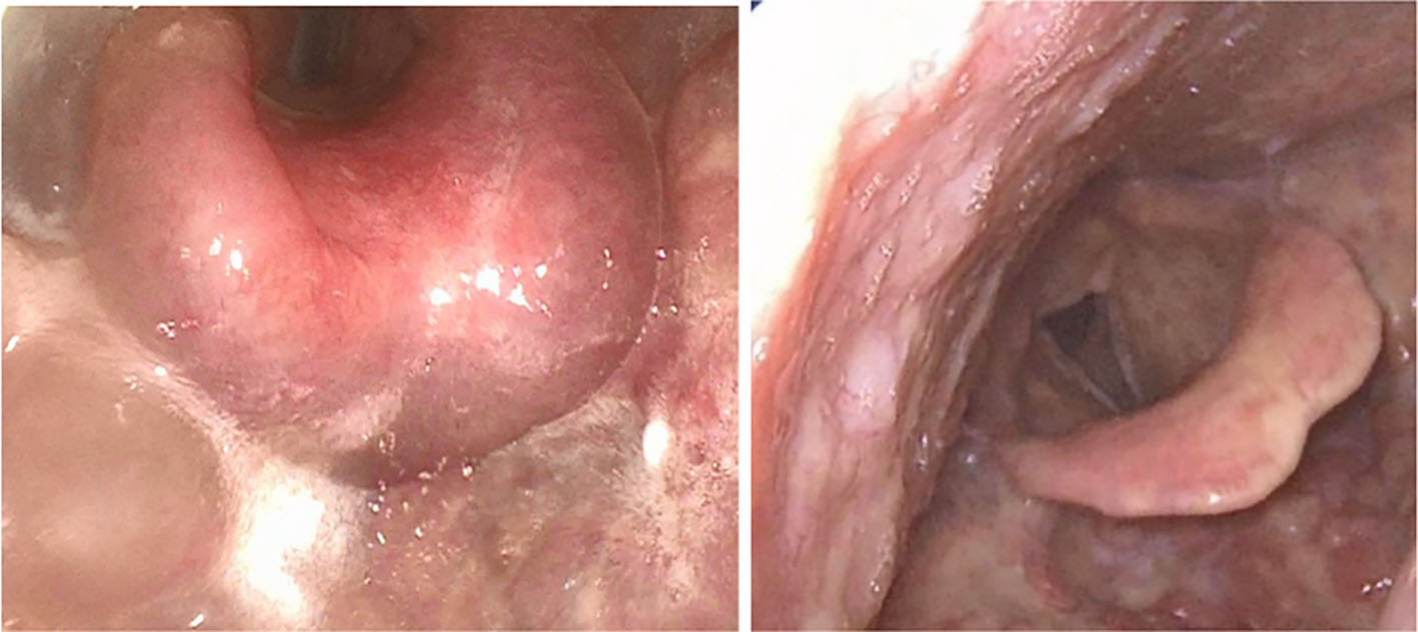
Life-threatening ACE inhibitor-induced angioedema with edematous swelling of the larynx and epiglottis (left) and after swelling subsides (right).

Currently, no approved treatment exists specifically for ACE-inhibitor-induced angioedema. However, a prospective, double-blind study involving 27 patients demonstrated significant improvement of laryngeal attacks with icatibant compared to treatment with glucocorticosteroids and antihistamines (55). Upper airway swelling is a common presentation in ACE-inhibitor-associated angioedema (see life-threatening angioedema of the epiglottis in **Figure 3**) and can also lead to airway obstruction and may be lethal. In suspected cases a If these conditions are suspected, ACE-inhibitors or sartans must be discontinued immediately, which prevents recurrence but does not affect the current episode. Physicians should monitor patients closely—preferably in an intensive care setting—and avoid delaying intubation or cricothyroidotomy if the airway deteriorates.

### Future treatments for bradykinin-mediated angioedema

Donidalorsen is an investigational RNA-targeted therapy designed to reduce prekallikrein expression, thereby lowering bradykinin-mediated hereditary angioedema (HAE) attacks. In the Phase III trial, conducted over 24 weeks, s.c. administration of 80 mg every 4 weeks reduced attack rates by 81% compared to placebo, while the 8-week regimen produced a 55% reduction (ARR 4-week: 1.82; ARR 8-week: 1.24). Patient-reported outcomes, including AE-QoL scores, improved alongside the reduction in attack frequency (56). While these results demonstrate strong efficacy in a controlled setting, its effectiveness in a real-world setting is unknown. Comparative effectiveness versus injectable prophylactic agents such as lanadelumab or garadacimab has not been performed. Adverse events reported were generally mild, primarily injection-site reactions and transient laboratory abnormalities. Overall, donidalorsen shows promise as a less frequent, RNA-targeted prophylactic option, but its positioning relative to existing therapies remains unclear, and long-term data are not yet available.

Deucrictibant is an oral bradykinin B2 receptor antagonist for on-demand treatment of HAE. In a Phase II, double-blind, randomized, placebo-controlled, crossover study, immediate-release capsules accelerated symptom resolution, with median time to end of progression of 25–26 minutes across doses (10–30 mg) compared with 20 hours for placebo (ARR 10 mg: 49.0%; ARR 20 mg: 59.9%; ARR 30 mg: 64.1%). Patient-reported outcomes were not extensively reported, limiting insights into broader quality-of-life effects. Adverse events were generally mild, including headache and upper respiratory symptoms. While these early results are promising, the evidence base remains moderate due to the small sample size and lack of phase III confirmation. A phase III study is currently being performed with results expected at the end of 2025. Comparative effectiveness versus other oral or injectable on-demand therapies, cost-effectiveness analyses, and real-world performance—particularly in severe laryngeal or pharyngeal attacks—are currently unknown. Ongoing larger trials and head-to-head studies are required to define the clinical role of deucrictibant and its potential advantages over existing therapies (57, 58).

Phase 2 studies have been performed evaluating the use of an extended-release version of deucrictibant as a once daily oral prophylactic treatment. These demonstrated an 84.5% reduction in attacks with a 40mg dosage in comparison to placebo. A further phase 3 study is ongoing to evaluate its efficacy (59).

Navenibart is a long-acting, YTE-modified human IgG1 monoclonal antibody that selectively inhibits plasma kallikrein. It is administered subcutaneously, with extended dosing intervals under investigation, including every three or six months. Interim and final topline results from Phase 1b/2 trials describe marked efficacy, with mean monthly attack rate reductions in the 90–95% range at six months and up to two-thirds of patients remaining attack-free in certain cohorts. The treatment was generally well tolerated, with adverse events primarily mild—most commonly injection-site reactions—and no emergent safety concerns identified to date. A pivotal Phase 3 program has been initiated, but confirmation of efficacy and safety in larger, long-term studies remains essential (60, 61).

Genome-targeted prophylaxis: NTLA-2002 is a one-time, *in vivo* CRISPR/Cas9 therapy delivered via lipid nanoparticles that selectively enter hepatocytes. Within these liver cells, it disrupts the KLKB1 gene, which encodes prekallikrein. By reducing kallikrein production, downstream bradykinin generation is suppressed, targeting the central mediator of angioedema attacks. This approach is designed to achieve a durable effect after a single infusion.

Early clinical data are striking but remain preliminary. In the Phase 2 trial, 11 patients treated with a single 50 mg dose experienced an average ~95% reduction in attack frequency over the 16-week evaluation period, with eight remaining completely attack-free. Patient-reported outcomes, including AE-QoL scores, also improved meaningfully in parallel with symptom control. Longer-term follow-up from the Phase 1/2 cohorts now extends beyond 20 months with sustained kallikrein suppression and attack reductions of 97–99%. Described side effects included mild infusion-related reactions and transient laboratory abnormalities, and no late toxicities have yet been observed (62, 63).

Despite these encouraging results, the evidence base is limited to small, early-phase studies compared with placebo. Importantly, the long-term safety of permanent gene editing is unknown, and off-target or structural genomic changes remain a concern. These agents can be administered as infrequently as every four weeks, demonstrate robust efficacy with >80–90% attack reductions, and are supported by a much larger safety database with generally mild side effects. The balance of benefit versus risk for NTLA-2002 is less clear, especially in younger patients or those with milder disease. This raises ethical questions about offering irreversible genome-editing therapies when highly effective and reversible prophylactic agents with established safety are already available. Larger randomized trials, longer surveillance, and careful ethical evaluation will be essential before the role of NTLA-2002 in routine care can be defined.

## Summary

Angioedemas, including urticaria and bradykinin-mediated forms, are clinically diverse and, in some cases, potentially life-threatening conditions. In both entities, angioedema may present as the main or only symptom, which makes thorough clinical evaluation essential. Differentiating between histamine-mediated and bradykinin-mediated angioedema is particularly important, as treatment strategies differ significantly.

In hereditary angioedema (HAE), accurate diagnosis is often delayed due to its rarity and overlapping features with other forms of angioedema. In certain cases, genetic analysis can support the diagnosis, particularly in patients without a clear family history or with normal C1-INH levels.

The growing number of novel therapies has significantly improved disease control and quality of life for many patients. However, it also adds complexity to clinical decision-making for both patients and physicians. Treatment decisions should be individualized based on attack frequency, severity, and patient preference. For rarer subtypes such as acquired angioedema or HAE with normal C1-INH, therapeutic strategies are often extrapolated from HAE data and may require a case-by-case approach.

Guidelines have yet to determine whether short-term prophylaxis is necessary in patients already receiving effective long-term prophylaxis. Additional short-term prophylaxis may still be warranted in situations with a high risk of provoking attacks. The practicality of oral on-demand treatments remains uncertain, particularly during severe pharyngeal or laryngeal episodes that can impair swallowing.

Current decision-making will therefore need to rely on real-world data, which remains limited. Emerging gene editing therapies suggest a potential long-term shift toward longer-lasting or even curative treatment. Ongoing research and increased awareness are essential to close diagnostic gaps, improve outcomes, and ensure that advances in therapy can safely reach all affected patient groups.
